# Head Start educators’ professional well-being and their turnover intentions: the moderating role of perceived workplace discrimination

**DOI:** 10.3389/fpsyg.2025.1642654

**Published:** 2025-10-16

**Authors:** Xiangyu Zhao, Sooyeon Byun, Lieny Jeon

**Affiliations:** ^1^Department of Education Leadership, Foundations and Policy, University of Virginia School of Education and Human Development, Charlottesville, VA, United States; ^2^Department of Child and Family Studies, Yonsei University, Seodaemun-gu, Republic of Korea

**Keywords:** early care and education, Head Start, compassion satisfaction, secondary traumatic stress, emotional exhaustion, workplace discrimination, turnover intention

## Abstract

High turnover is a critical challenge for Head Start programs. This study aims to understand how professional well-being and workplace factors are related to turnover intentions within Head Start educators. Utilizing hierarchical linear modeling with a sample of 304 educators, the study examined how positive aspects (i.e., compassion satisfaction) and negative aspects (i.e., secondary traumatic stress and emotional exhaustion) of professional well-being, along with perceived workplace discrimination, are associated with their turnover intentions (i.e., intention to leave the profession, program, or position). The findings demonstrated that compassion satisfaction, emotional exhaustion, and workplace discrimination were significantly associated with turnover intentions. The study also descriptively examined the specific reasons behind these intentions, which included poor benefits and compensation, classroom management stress, and a lack of advancement opportunities. These findings suggest the need for interventions and policies to enhance educators’ professional well-being, address workplace discrimination, and improve working conditions to retain qualified Head Start educators.

## Introduction

High-quality early care and education (ECE) relies on the availability of dedicated and professional educators (Jeon et al., 2014). However, due to a combination of factors, including demanding workloads, low compensation, stressful work environment, and low social recognition, ECE educators face significant challenges that are associated with high turnover rates (Jeon and Wells, 2018; Kwon et al., 2022a; Wells, 2015). ECE educators’ turnover may be negatively associated with the development of both children and ECE programs. For example, ECE educators’ turnover can disrupt the attachment building of children and make it difficult for families to adjust to new caregivers (Bryant et al., 2023; Kwon et al., 2022a). For ECE programs, turnover creates additional effort and financial costs associated with constantly recruiting, hiring, training, and mentoring high-quality teachers (Bryant et al., 2023). Thus, it is important to understand the factors that may explain teachers’ turnover intentions.

The high turnover issue is particularly pronounced among Head Start educators working with children and families who benefit most from high-quality ECE (Wells, 2015). Distinct from other ECE programs, Head Start programs provide free federally funded services specifically for children from birth to five who live in poverty in the United States (U.S. Department of Health and Human Services, 2025). Head Start offers a two-generational approach aimed at strengthening children’s development and health as well as improving the well-being of their families (U.S. Department of Health and Human Services, 2025). Research suggests that Head Start educators face demanding work environments with unique challenges. They tend to work with children experiencing maltreatment and trauma (Data Resource Center for Child and Adolescent Health, 2022). As a result, these educators often need to manage high levels of children’s challenging behaviors (Kwon et al., 2022a). Additionally, as federally monitored programs, Head Start requires educators to complete more paperwork compared to other ECE programs (Li et al., 2025); thus, educators may perceive heavier administrative burdens, which reduces their professional commitment and decision to stay (Wells, 2015). Moreover, there is limited knowledge regarding specific turnover intentions of Head Start educators, including leaving the ECE field, leaving their current program, or leaving their present position. These turnover intentions may relate to their distinct motivations and performances (Grant et al., 2019). The current study aims to provide a better understanding of factors related to Head Start educators’ various types of turnover intentions.

According to the Ecological Model of Holistic Early Childhood Workforce Well-being, educators’ professional well-being is an important aspect of ECE educators’ well-being and can impact their practice, behaviors, and retention (Jeon et al., 2022). As for Head Start educators, secondary traumatic stress (STS), emotional exhaustion, and compassion satisfaction are critical components of their professional well-being that may be related to their reduced commitment. Due to their care for children in poverty who are often exposed to trauma (Brown, 2016), Head Start educators are particularly susceptible to STS, which are the natural, consequent behaviors and emotions resulting from exposure to details of another person’s trauma (Bride et al., 2004). Additionally, because of the heavy administrative burdens and the challenges of managing children’s behavioral issues, Head Start educators are more likely to experience emotional exhaustion than other educators (Kwon et al., 2022a). The heightened levels of STS and emotional exhaustion may be related to their reduced professional commitment and increased intent to leave (Brown, 2016; Kwon et al., 2022a). However, the picture is not entirely negative. Despite the challenges, many ECE educators find the work rewarding as they support young children’s growth and development (McDonald et al., 2018). This suggests that their compassion satisfaction, a sense of fulfillment derived from helping children and families in need (Stamm, 2009), can potentially strengthen Head Start educators’ commitment, and thereby foster their intention to stay in their jobs.

In addition, the study investigates the role of teacher-perceived working environment, specifically workplace discrimination, in the associations between professional well-being and turnover intentions. The Early Childhood Professional Well-Being Framework suggests that organizational culture, including experiences of discrimination, can negatively impact an educator’s well-being (Gallagher and Roberts, 2022), potentially leading to decreased job commitment and a higher likelihood of leaving. Additionally, the intersectionality theory posits that individuals can simultaneously experience oppression and privilege because of their various social identities, which may include aspects of race, sex, gender, age, ability status, socioeconomic status, and religious identity (Dhanani and El-Farr, 2016; Hill Collins and Bilge, 2020). For Head Start educators who are disproportionately women of color, earn low salaries, and are often devalued in their roles (Bacon, 2019; Boyd, 2013), it is likely that their perceived discrimination may be derived from these different sources of oppression. While research in other fields has established a link between perceived discrimination, well-being, and turnover intentions (Dhanani et al., 2018; Sert Ozen et al., 2021), the impact of workplace discrimination on ECE professionals is largely unknown. Given the historical marginalization and low social recognition of the ECE workforce (McLean et al., 2021), it is critical to examine this specific environmental factor within their professional lives.

This study, guided by the Ecological Model of Holistic Early Childhood Workforce Well-being Framework (Jeon et al., 2022) and the Early Childhood Professional Well-Being Framework (Gallagher and Roberts, 2022), examines the relationships between Head Start educators’ professional well-being (i.e., STS, emotional exhaustion, compassion satisfaction) and perceived workplace discrimination, and their intention to leave the profession, program, or position. In addition to the exploration of the three primary turnover intentions, the study also descriptively examined the specific reasons underlying these intentions. This comprehensive approach can inform more targeted interventions and policies to reduce turnover, retain qualified ECE professionals, and ultimately benefit the learning experiences of young children.

### Early childhood educators’ turnover intentions

The ECE workforce turnover rate is alarmingly high, with 25 to 50% of teachers annually leaving their jobs (Bassok et al., 2021; Jeon and Wells, 2018; Wells, 2015). Even within highly-resourced networks of schools, teacher turnover rates exceeded 30% per year (Bassok et al., 2021). Studies specifically focused on Head Start teachers have found that nearly 34 to 36% leave their programs each year (Bassok et al., 2021; Wells, 2015). These alarming turnover rates are negatively related to children’s social and emotional development and disrupt family-provider relationships (Bryant et al., 2023; Horm et al., 2018). In particular, the loss of highly skilled and experienced teachers can interrupt children’s attachment and relationship-building processes with educators, negatively relating to the development of their social, emotional, and executive functioning skills (Graziano et al., 2016; Horm et al., 2018). Additionally, the turnover of teachers requires the remaining staff to cover additional duties, adjust routines, and invest time and energy in building connections with new staff. This can create a stressful work environment for the remaining staff and is potentially associated with their own reduced professional engagement (Bryant et al., 2023). Moreover, research has shown that high teacher turnover is more prevalent in schools with limited resources and those located in marginalized communities (Darling-Hammond, 2010). These schools often work with a high proportion of students from disadvantaged backgrounds, and as a result, teachers’ turnover can exacerbate educational inequities.

Given the potential negative impacts of high turnover among ECE educators, it is crucial to investigate the factors related to the proximal precursor of turnover-turnover intentions (McInerney et al., 2015). Empirical research also suggests that employees’ turnover intentions are correlated with their turnover behaviors, although the correlation may vary by demographic characteristics (Kellerer and Süß, 2025). Exploring turnover intentions may provide insights into how to better support educators in order to reduce their actual turnover rates. In this study, we distinguish between three types of teacher turnover intentions: intention to leave the profession, intention to leave a current program, and intention to leave a current position. This distinction is important because the experiences and motivations behind these intentions may differ. For instance, teachers who plan to leave the ECE field entirely may be dissatisfied with the low social recognition and poor compensation across the overall ECE profession (Bassok et al., 2021). Alternatively, teachers who intend to leave their current program may be motivated by a lack of sense of community and program-level support (Grant et al., 2019), seeking opportunities in programs with better resources and a more positive work environment. Additionally, teachers who consider leaving their current position may seek advancement to a leadership role within the same program (Crawford et al., 2010). Distinguishing between these turnover intentions can provide valuable insights for program leaders and policymakers, allowing them to tailor support strategies for educators based on their specific motivations.

While poor salaries and benefits are recognized as significant factors related to high teacher turnover rates (McLean et al., 2021), they are not the only determining factors. Teachers’ personal characteristics, states of well-being, and their perception of their work environments are also critical factors in their attitudes towards their jobs (Jeon and Wells, 2018; Thorpe et al., 2020). Given the high standards and the specialized knowledge and skills required to work with children in poverty (Office of Head Start, 2022), the professional experiences and well-being of Head Start educators may differ from those of people working in other ECE settings. Additionally, Head Start educators are disproportionately female and of color, both groups that face discrimination based on their professional and personal identities during their work (Sparks, 2019). These discriminatory experiences may also be associated with teachers’ intentions to leave their jobs. The current study examines these factors in relation to teachers’ intent to leave. Furthermore, to provide more personalized support, this study also investigates the underlying specific reasons that drive Head Start educators to consider leaving their positions using an open-ended question.

### Secondary traumatic stress and its associations with turnover intentions

STS is defined as the natural, consequent behaviors and emotions resulting from exposure to details of another person’s trauma (Bride et al., 2004). The Substance Abuse and Mental Health Services Administration (2023) reported that over two-thirds of children in the U.S. went through at least one traumatic experience by age 16. Educators who work with these children may experience high levels of STS and related symptoms (Berger and Nott, 2024). In fact, studies have shown that approximately 38 to 43% of teachers report experiencing high levels of STS (Berger and Nott, 2024; Ormiston et al., 2022).

Although STS is understudied within the ECE field, studies suggest that STS is more prevalent among teachers working in underserved schools and schools with high populations of marginalized and racially and ethnically diverse students (Christian-Brandt et al., 2020; Denham, 2018; Ormiston et al., 2022). Likewise, Head Start educators, who work with children from low-income families and marginalized communities, are particularly vulnerable to STS, due to their frequent exposure to children’s traumatic experiences, such as violence and crime (Rankin, 2021). These untreated STS experiences may be adversely related to educators’ personal and professional experiences, including increased symptoms of depression, increased conflicts with children, and reduced job satisfaction (Caringi et al., 2015), which further increase the risk of turnover. Research in K-12 context also demonstrates that STS is potentially associated with educators’ turnover intention. For instance, a study found that 75% of educators working with trauma-affected youth considered leaving the teaching profession through career changes, retirement, or transitioning to other schools (Caringi et al., 2015). Similarly, teachers working in resource-limited schools with higher minority populations face increased risks of STS and teacher attrition (Reilly et al., 2025). These findings are particularly relevant to Head Start educators, who often work in similar settings and experience high levels of STS. Beyond psychological symptoms, STS can also relate to individuals’ physical symptoms, such as respiratory problems, muscular discomfort, nightmares, and insomnia (Jang et al., 2023), which Head Start educators report at high levels (Jeon et al., 2020). These physical and psychological symptoms may deplete educators’ emotional and physical resources and explain the potential associations between STS and educators’ absenteeism and turnover intention (Rankin, 2021).

### Emotional exhaustion and its associations with turnover intentions

In addition to STS, emotional exhaustion is another prevalent occupational hazard among educators (Brown, 2016). Emotional exhaustion, a state of depletion of both emotional and physical dimensions from job demands (Cropanzano et al., 2003), can reduce individuals’ psychological engagement in their job (Maslach and Jackson, 1981). The experience of emotional exhaustion among ECE professionals is concerning, as teacher emotional exhaustion has been linked to both job attitudes (Han et al., 2021) and educational practices (Han et al., 2021). Research has demonstrated that early childhood educators experiencing higher levels of emotional exhaustion exhibit reduced commitment and comfort in providing sensitive support for children’s social–emotional learning (Hoglund et al., 2015). Moreover, children with emotionally exhausted teachers are more likely to display lower academic achievement and school engagement compared to their peers (Hoglund et al., 2015).

Emotional exhaustion is one of the critical aspects of teachers’ professional well-being that is associated with teachers’ turnover intentions (Schaack et al., 2020). A study of 273 early childhood educators in Colorado found that those who were emotionally exhausted were more likely to leave their jobs (Schaack et al., 2020). Similarly, another study reported that teachers with higher emotional exhaustion tend to want to leave the profession (Grant et al., 2019). While the connection between teachers’ emotional exhaustion and their intent to leave has received increasing attention, only a few studies specifically examined it among Head Start educators. Head Start educators face unique challenges at workplace, such as managing children’s challenging behaviors, handling excessive paperwork, and fulfilling the dual responsibility of providing support for children and building relationships with families to achieve the two-generation mission of Head Start programs (Kwon et al., 2022a; Wells, 2015). Despite the challenges, the relationships between Head Start educators’ emotional exhaustion and their specific turnover intentions, whether it be leaving the ECE profession entirely, leaving their current program, or leaving their current position, remain unclear. This study aims to address this gap by examining these distinct turnover intentions in relation to emotional exhaustion among Head Start educators.

### Compassion satisfaction and its association with educators’ turnover intentions

While the stress and demands of ECE are related to burnout and STS, many educators also find the work deeply rewarding (McDonald et al., 2018). This sense of fulfillment can be termed as compassion satisfaction, which arises from the pleasure and gratification derived from helping others and contributing to a larger purpose (Stamm, 2009). Individuals experiencing compassion satisfaction often report enhanced self-efficacy, a sense of invigoration, and the belief that their actions can make a positive impact on the world (Stamm, 2009). A qualitative study by Fleckman et al. (2022) explored the experiences of compassion satisfaction among teachers. They found that teachers experienced compassion satisfaction through positive feelings associated with helping students, witnessing their academic and social–emotional growth, experiencing a strong sense of professional self-efficacy, feeling connected to a supportive community, and cherishing the relationships they share with students and families (Fleckman et al., 2022).

While research specifically examining compassion satisfaction among ECE educators is limited, evidence from other fields suggests potential associations between compassion satisfaction and turnover intentions. For instance, in the medical sector, studies revealed that compassion satisfaction, compassion fatigue, and burnout collectively accounted for 20% of turnover intention among medical care workers (Ariapooran et al., 2021). Similarly, research among clinical social workers found that individuals who report low compassion satisfaction tend to perceive heightened personal distress, which is potentially related to the decision to leave the profession (Thomas, 2013). A study among elementary school teachers also suggests that low compassion satisfaction in conjunction with high burnout is associated with teachers’ increased intent to leave the profession (Christian-Brandt et al., 2020).

Many ECE educators report staying in their jobs due to the intrinsic motivation derived from making a positive difference in children, families, and society (McDonald et al., 2018). Interestingly, research suggests that teachers working in high-poverty schools perceived higher levels of compassion satisfaction compared to those in more affluent settings (Reilly et al., 2025). This could be particularly true for Head Start educators working with children in poverty, since compassion satisfaction may be a factor motivating them to stay in their job (Brown, 2016). A study during the COVID-19 pandemic also suggests that Head Start educators reported higher levels of compassion satisfaction compared to employees working in healthcare on average (Jeffrey et al., 2022). Head Start educators experience a combination of fulfillment from supporting vulnerable children and stress due to the increased likelihood of these children experiencing trauma and demonstrating challenging behaviors (Kwon et al., 2022a). Therefore, this study aims to simultaneously examine these three professional well-being factors (i.e., compassion satisfaction, emotional exhaustion, and STS) in order to better understand Head Start educators’ professional experiences.

### Moderating role of workplace discrimination

In addition to professional well-being, the working environment also plays a critical role in educators’ turnover intention (Grant et al., 2019). Workplace discrimination is an understudied topic in early childhood educators’ work environments, which is characterized as the unjust and negative treatment of employees or prospective job candidates, based on personal attributes that are not relevant to their job performance (Chung, 2001). Early childhood educators often experience societal bias and discrimination regarding their profession (Bacon, 2019; Boyd, 2013). This is evidenced by stereotypes that dismiss early childhood educators as only “babysitters” rather than skilled professionals (Bacon, 2019). Consequently, educators struggle for social recognition and perceive themselves as undervalued by society as a whole (Quinones et al., 2021). This societal bias and discrimination are also related to the ECE workforce being some of the lowest-paid workers in the United States, even though their work enables parents to participate in the workforce and promote the healthy development of young children (McLean et al., 2021).

Unfortunately, discrimination also exists within the ECE profession itself. Although the workforce is predominantly female and racially diverse (Park et al., 2015), this diversity does not reflect equal opportunities. Research conducted within 14 U.S. cities found that, among ECE teachers with identical model resumes, Black and Hispanic applicants received significantly fewer interview requests than White applicants with similar qualifications and backgrounds (Boyd-Swan and Herbst, 2019). Additionally, Black ECE teachers are more likely to work with infants and toddlers, which are positions that pay lower wages than those who teach preschool-age children. In contrast, White teachers are more likely to work in school-sponsored ECE programs, which provide higher pay and benefits (Schilder and Curenton, 2021). Furthermore, wage disparities exist even within the diverse female ECE workforce. A national study found that women of color, including Black, Hispanic/Latina, and Asian, earn the lowest average hourly wages (Liu et al., 2025). Additionally, while Black and Hispanic/Latino men in ECE have higher average wages than women of color, they still earn less than White women (Liu et al., 2025).

The combined effects of societal bias and internal discrimination within the ECE profession raise concerns about its potential adverse associations with educators’ well-being and retention. While no research has directly examined the relationships between perceived discrimination and turnover intentions among ECE educators, evidence from other fields suggests a connection between these factors. Studies in the business field have found that employees who experience discrimination are more likely to report lower job satisfaction and express a desire to leave their jobs (Elçi et al., 2021; Qu et al., 2019). Research among staff in higher education institutions also demonstrates that when individuals suppress group identity and perceive high levels of workplace discrimination, they tend to think about quitting their jobs (Madera et al., 2012). This evidence suggests that perceived discrimination may play a significant role in turnover intentions among ECE educators as well. Recognizing and understanding this relationship is crucial for fostering a more equitable and inclusive ECE profession.

The Early Childhood Professional Well-Being Framework posits that organizational culture, including the experience of discrimination at work, can be harmful to one’s professional well-being (Gallagher and Roberts, 2022), which ultimately may impact the associations with job commitment and engagement. Similarly, self-determination theory suggests that individuals’ perceived working environment is related to motivation for their practices at work (Ryan and Deci, 2000). Therefore, it is possible that workplace discrimination interacts with the associations between educators’ well-being and their turnover intentions. For instance, when educators face frequent workplace discrimination, the role of STS and emotional exhaustion in their job commitment may be stronger. This may be because the pressure from a hostile work environment compounds upon existing feelings of low well-being, ultimately pushing them towards wanting to leave. Conversely, educators who perceive a more inclusive and just work environment where discrimination is low, may experience a buffering effect. In such cases, even if they experience emotional exhaustion, STS, and low compassion satisfaction, their desire to leave might be buffered by their overall satisfaction with the workplace culture.

In addition to examining the potential associations between well-being and organizational factors and educators’ professional attitudes, it is also critical to understand the specific reasons why educators considered leaving their jobs from their perspective. The high turnover rates of Head Start educators and their potential negative impacts on children’s and program’s development are well documented (Wells, 2015). However, the literature mainly discussed financial hardships and demanding workloads as the main reasons for ECE educators’ turnover intentions without comprehensively investigating other potential reasons from Head Start educators’ perspectives (McLean et al., 2021; Zhao and Jeon, 2024). Therefore, we attempt to understand the specific and nuanced reasons why Head Start educators consider quitting their jobs, which can better inform the improvement of the Head Start environment.

## The present study

Head Start educators’ alarming turnover rate is related to the development of children in most need (Wells, 2015). According to evidence from existing literature and the theories, professional well-being and perceived workplace discrimination potentially play critical roles in Head Start turnover intentions (Jeon et al., 2022; Ryan and Deci, 2000; Schaack et al., 2020; Sprang et al., 2011). However, the relationships among these factors have not been comprehensively examined within Head Start educators. The current study aims to bridge the gap by answering the following questions.

1) What are the associations between Head Start educators’ professional well-being (e.g., STS, emotional exhaustion, compassion satisfaction), their perceived discrimination at the workplace, and their intent to leave their current position, program, and profession?

*H1a*: When Head Start educators experience a higher level of STS, they are more likely to consider leaving their profession, program, and position.

*H1b*: When Head Start educators experience a higher level of emotional exhaustion, they are more likely to consider leaving their profession, program, and position.

*H1c*: When Head Start educators perceive higher compassion satisfaction, they are less likely to consider leaving their profession, program, and position.

*H1d*: When Head Start educators perceive more frequent workplace discrimination, they are more likely to consider leaving their profession, program, and position.

2) To what extent does workplace discrimination moderate the associations between professional well-being and intent to leave?

*H2a*: The positive association between STS and turnover intentions will be stronger for teachers experiencing more frequent workplace discrimination.

*H2b*: The positive association between emotional exhaustion and turnover intentions will be stronger for teachers experiencing more frequent workplace discrimination.

*H2c*: The negative association between compassion satisfaction and turnover intentions will be weaker for teachers experiencing more frequent workplace discrimination.

3) What are the specific underlying reasons why Head Start educators consider leaving their jobs?

## Methods

### Participants

This study involved a survey of 304 educators across 54 Head Start sites located in two Mid-Atlantic states in the United States. These educators were recruited as part of a professional development (PD) training, specifically designed for enhancing the holistic well-being of Head Start educators. We only used pre-test data in the current study. Table 1 presents the demographic characteristics of the participants. Among those educators who completed the pre-test survey, 35.6% were lead teachers, 30.7% were assistant teachers, and 19.5% were in various administrative roles (e.g., program directors, family service coordinators, education coordinators), and 14.1% were in other ECE educator roles (e.g., substitute teacher, teacher aid, and home visitors). Around 96.2% of survey respondents were female. The ethnic composition of the participants was primarily Black/African American, non-Hispanic (56.1%), followed by Hispanic (21.3%), and White, non-Hispanic (18.1%). The average age of participants was approximately 40.7 years, with an average of 11.1 years of experience in ECE. Regarding the educational attainment among the participants, 62.2% of them held an associate degree or higher, and 41.1% had a bachelor’s degree or above. In terms of salary, the majority (64.5%) reported annual earnings ranging from $20,000 to $50,000.

**Table 1:** Participants characteristics.

Variable	*N*	Mean/%	*SD*	Range
Age	237	40.67	12.54	19–73
Female	278	96.19%	-	0–1
Race/ethnicity	287			
White, non-Hispanic	52	18.12%	-	0–1
Black, non-Hispanic	161	56.10%	-	0–1
Hispanic	61	21.25%	-	0–1
Others	13	4.53%	-	0–1
Married or cohabitating	104	36.49%	-	0–1
Educational attainment	304			
Less than high school, no GED	2	0.66%	-	0–1
High school diploma or GED	113	37.17%	-	0–1
Associate’s degree	64	21.05%	-	0–1
Bachelor’s degree	92	30.26%	-	0–1
Master’s degree or above	33	10.85%	-	0–1
Educator role	303			
Lead teacher	108	35.64%	-	0–1
Assistant teacher	93	30.69%	-	0–1
Administrative roles	59	19.47%	-	0–1
Other ECE educator roles^a^	43	14.14%	-	0–1
Annual salary	260			
$10,000 or less	11	3.89%	-	0–1
$10,001 to $20,000	19	6.71%	-	0–1
$20,001 to $30,000	69	24.38%	-	0–1
$30,001 to $40,000	72	25.44%	-	0–1
$40,001 to $50,000	42	14.84%	-	0–1
$50,001 to $60,000	24	8.48%	-	0–1
$60,001 to $70,000	16	5.65%	-	0–1
$70,001 or more	7	2.47%	-	0–1
Years of experience in ECE	299	11.06	9.47	0–44
Years of experience in current Head Start	285	5.34	6.31	0–37

Total *N* = 304. GED = Graduate Equivalency Degree, ECE = Early Care and Education.

a Other ECE educator roles included substitute teacher, teacher aid, family resource worker, home visitor office assistant, etc.

### Procedures

The study received ethical approval from the University Institutional Review Board. Data were collected in 2023 and 2024 for a larger project, designed to provide a PD program, Well-Being First, for Head Start educators. Head Start grantees and leaders interested in participating in the PD program were contacted by the research team. After the programs were assigned to either a treatment group or a control group, the research team received a roster of all staff members in each program, including lead teachers, assistant teachers, and administrators. Then, the research team sent a pre-test survey link along with the informed consent form to each individual. All pre-test data used in this study were collected prior to the first training session. The survey asked participants’ demographic characteristics, well-being, perceptions of their work environments, and professional commitment and attitudes. Out of 530 participants who received the survey links, 309 completed the survey, resulting in a response rate of 58.3%. Given that the current study aims to focus on educators, we excluded the data from 5 participants whose job titles were related to kitchen and data analysis, which might lack direct relevance to classroom teaching. Each participant who completed the survey was compensated with $25 as an appreciation.

### Measures

#### Turnover intentions

This study assessed three types of turnover intentions among Head Start educators: leaving the ECE profession, leaving their current program, and leaving their current position. To measure their intention to leave the ECE profession, participants rated their agreement with the statement “Within the next 12 months, I intend to continue as an early childhood educator” on a scale from 1 (*Strongly Disagree*) to 5 (*Strongly Agree*; Buettner et al., 2016). We reverse-coded this item, and higher scores indicated a stronger intention to leave the profession. The intention to leave the current program was measured by the statement “Within the next 12 months, I plan to remain at my current program,” using the same 5-point scale (1 = *Strongly Disagree*, 5 = *Strongly Agree*; Buettner et al., 2016). We reverse-coded this item, and higher scores suggested a higher likelihood of leaving the program. To assess intentions to leave their current position, participants answered “How many more years do you plan to be in your present position within your program?” with options from “1 = *2 years or less*” to “4 = *10 or more years*.” We reverse-coded this item, and higher scores indicated a stronger desire to leave their position sooner. We used three clear, direct, and single-item questions for each type of turnover intention to allow participants to reflect on each specific intention without conflating the different levels of potential turnover. Additionally, research shows that single-item measures can be valid and reliable when measuring concrete and unambiguous constructs (Nagy, 2002; Siebens et al., 2015). Our items are aligned with the literature and have been used in previous research projects and related studies examining educators’ professional attitudes (e.g., Zhao et al., 2025).

#### Secondary traumatic stress

We adopted the Secondary Traumatic Stress Scale developed by Stamm (2009) to measure educators’ STS. The scale consists of nine items, with a high internal reliability (Cronbach’s *α* = 0.91) in our sample. Educators rated items reflecting their exposure to proximal traumatic experiences on a 5-point Likert scale (1 = *Never*, 5 = *Very Often*). Example items include “I think I am affected by the traumatic stress of the children and families I educate and care for.” The sum of the scores was calculated to represent the educators’ level of STS. A higher score indicates a higher level of STS.

#### Emotional exhaustion

We adopted Maslach’s Burnout Inventory to measure Head Start educators’ emotional exhaustion (Maslach and Jackson, 1981). Participants rated eight items to report the frequency of experiencing emotional exhaustion, using a 7-point Likert scale (1 = *Never*, 7 = *Everyday*). Example items include “I feel emotionally drained from my work.” The scale demonstrated good internal reliability in the current sample (Cronbach’s alpha = 0.90). The total score indicates participants’ emotional exhaustion level, with higher scores representing more frequent emotional exhaustion.

#### Compassion satisfaction

Educators’ compassion satisfaction was measured using the Professional Quality of Life Scale (ProQOL) subscale (Stamm, 2009). This subscale, with an internal reliability of 0.90 in the current sample, consists of 10 items assessing job satisfaction and fulfillment from serving in a helping role. Ratings were on a 5-point Likert scale (1 = *Never*, 5 = *Very Often*). Example items include “I feel invigorated after working with the children and families I care for.” The higher average score of these items reflects higher levels of compassion satisfaction.

#### Workplace discrimination

Perceived workplace discrimination was measured using the Workplace Discrimination Scale (Kwon et al., n.d.). This scale includes six items rated on a 5-point Likert scale (1 = *Strongly Disagree*, 5 = *Strongly Agree*), with an acceptable internal reliability of 0.72 in the current sample. Participants reported the characteristics of their work environment. Examples include “I am treated unfairly at work based on my identity or appearance.” The average scores indicate the educators’ perception of workplace discrimination, and higher average scores suggest more perceived discrimination.

#### Reasons for turnover intentions

To understand the specific reasons behind educators’ intentions to leave their jobs, participants who indicated a desire to leave within the next two years were asked to specify their reasons in the survey. The participants were given a predefined list of 13 options in the online survey. These 13 options were informed by prior literature and research (e.g., McKelvey et al., 2018), adopted with expert consultation, and piloted for clarity in the pilot study of this project. Participants also had the opportunity to provide alternative reasons via an open-ended “Other” response option. This approach ensured the collection of relevant data from those considering turnover.

#### Covariates

The present study controlled for educators’ demographics and other characteristics that may be related to their turnover intentions. Specifically, we controlled for educators’ years of working experience, salary, current teaching position (“*Lead teacher*” as the reference category; “*Assistant teacher*”; “*Administrative roles*”; “*Other roles*”), race/ethnicity (“*White, non-Hispanic*” as the reference category; “*Black, non-Hispanic*,” “*Hispanic*,” and “*Other*”), educational attainment (1 = *Bachelor’s degree or higher*, 0 = *Others*).

### Analytic strategy

#### Missing data

Regarding the missing data in the outcome variables, there were 9.21% of missing data in educators’ intention to leave their profession, 10.20% of missing in intention to leave their current program, and 6.25% of missing in intention to leave their current position. In terms of the independent variables, there was no missing in STS, 6.91% of missing in emotional exhaustion, and 9.87% of missing in compassion satisfaction, 6.25% of missing in workplace discrimination. Among the covariates, 5.59% of missing in race/ethnicity, 1.64% of missing in years of working experiences, 0.33% missing in educator’s title, 6.91% missing in salary, and no missing in education attainment. We did not find statistically significant differences between participants with complete data and those with incomplete data regarding their professional well-being, perceived workplace discrimination, and turnover intentions. To address the issue of missing data, we employed the method of multivariate imputation by chained equations and generated 10 imputed datasets to capture the statistical uncertainty in cross-sectional data (Austin et al., 2021; Molenberghs and Kenward, 2017). In addition, we compared the results of the primary models from the imputed data to those from the complete-case analysis. The significance and patterns of the results remained consistent between both data sets, and we did not find any substantial differences in variance components, which suggested that the imputation did not substantially bias the variance estimates.

#### Data analysis

The data in the current study had a two-level nested structure, with educators (Level 1) nested in Head Start centers (Level 2). Given this nested structure, we built a two-level hierarchical linear modeling in Stata 16.0 to answer the first and second research questions. First of all, we analyzed unconditional baseline models without predictor variables. We analyzed the intraclass correlation coefficients (ICCs), which describe the ratio of the between-cluster variance to the total variance. In particular, we analyzed ICCs for three types of educators’ turnover intentions, including their intention to leave the ECE profession, their current Head Start program, or their current position.

In the multi-level analyses, we first included only control variables in the models to estimate the model fit and the explained variances of the baseline models. The model fit indices that were used include Deviance (Hardin and Hilbe, 2018), Akaike’s information criterion (AIC; Akaike, 1987), and Bayesian information criterion (BIC; Schwarz, 1978). Second, in the main effects models, we added STS, emotional exhaustion, compassion satisfaction, and workplace discrimination variables. We compared the main effects models with the baseline models that did not include control variables to calculate the total variances explained in each outcome variable. Then, we compared the main effects models with the baseline models only with control variables, to calculate the unique variance explained by STS, emotional exhaustion, and compassion satisfaction after accounting for the control variables. Next, we added the interaction terms between key independent variables (i.e., STS, emotional exhaustion, and compassion satisfaction) and perceived workplace discrimination to the main effects models. Additionally, during the model building process, we compared the models with and without random slopes across centers between the independent and dependent variables. In particular, we examined three distinct models for three types of turnover intentions (i.e., intention to leave the profession, program, and position). For each model, we tested all possible combinations of random slopes for the four key predictors. This resulted in 45 model comparisons with the random intercept only models. We also checked the convergence of these models during comparisons. It is noted that models allowing both random slopes and intercepts did not demonstrate better model fit compared to models with only random intercepts. We found that the Deviance, AIC, and BIC values of these random slopes models remained largely similar to the random intercept only models. Given that there was no meaningful improvement in model fit with random slopes, we decided to keep the more parsimonious random intercept-only models. Therefore, we chose models that allowed intercepts to vary and fixed the slopes in each model. The final main effect models are:

Level-1 model:


$$\begin{array}{l} Y_{\mathrm{ij}}=\beta_{0j}+\beta_{1j}\mathrm{ST}S_{\mathrm{ij}}+\beta_{2j}\mathrm{Em}o_{\mathrm{ij}}+\beta_{3j}\mathrm{Co}m_{\mathrm{ij}}+\\ \beta_{4j}\mathrm{Di}s_{\mathrm{ij}}+\beta_{5j}\mathrm{ST}S_{\mathrm{ij}}*\mathrm{Di}s_{\mathrm{ij}}+\\ \beta_{6j}\mathrm{Em}o_{\mathrm{ij}}*\mathrm{Di}s_{\mathrm{ij}}+\beta_{7j}\mathrm{Co}m_{\mathrm{ij}}*\\ \mathrm{Di}s_{\mathrm{ij}}+\beta_{8j}{\mathrm{cov}}_{\mathrm{ij}}+e_{\mathrm{ij}} \end{array}$$

Level-2 model:


$$\beta_{0j}=\gamma_{00}+\mu_{0j}$$

$$\beta_{1j}=\gamma_{10}+\mu_{1j}$$

$$\beta_{2j}=\gamma_{20}+\mu_{2j}$$

$$\beta_{3j}=\gamma_{30}+\mu_{3j}$$

$$\beta_{4j}=\gamma_{40}+\mu_{4j}$$

$$\beta_{5j}=\gamma_{50}+\mu_{5j}$$

$$\beta_{6j}=\gamma_{60}+\mu_{6j}$$

$$\beta_{7j}=\gamma_{70}+\mu_{7j}$$

$$\beta_{8j}=\gamma_{80}+\mu_{8j}$$

Combined model


$$\begin{array}{l} Y_{\mathrm{ij}}=\gamma_{00}+\gamma_{10}\mathrm{ST}S_{\mathrm{ij}}+\gamma_{20}\mathrm{Em}o_{\mathrm{ij}}+\gamma_{30}\mathrm{Co}m_{\mathrm{ij}}+\\ \gamma_{40}\mathrm{Di}s_{\mathrm{ij}}+\gamma_{50}\mathrm{ST}S_{\mathrm{ij}}*\mathrm{Di}s_{\mathrm{ij}}+\\ \gamma_{60}\mathrm{Em}o_{\mathrm{ij}}*\mathrm{Di}s_{\mathrm{ij}}+\gamma_{70}\mathrm{Co}m_{\mathrm{ij}}*\\ \mathrm{Di}s_{\mathrm{ij}}+\gamma_{80}{\mathrm{cov}}_{\mathrm{ij}}+\mu_{0j}+u_{1j}\mathrm{ST}S_{\mathrm{ij}}+\\ u_{2j}\mathrm{Em}o_{\mathrm{ij}}+u_{3j}\mathrm{Co}m_{\mathrm{ij}}+u_{4j}\mathrm{Di}s_{\mathrm{ij}}+\\ u_{5j}\mathrm{ST}S_{\mathrm{ij}}*\mathrm{Di}s_{\mathrm{ij}}+u_{6j}\mathrm{Em}o_{\mathrm{ij}}*\mathrm{Di}s_{\mathrm{ij}}+\\ u_{7j}\mathrm{Co}m_{\mathrm{ij}}*\mathrm{Di}s_{\mathrm{ij}}+u_{8j}{\mathrm{cov}}_{\mathrm{ij}}+e_{\mathrm{ij}} \end{array}$$

Where $Y_{\mathrm{ij}}$ represents educators’ turnover intentions (i.e., intention to leave the ECE profession, current Head Start program, or current position) for educator i in center j; Cov in Level 1 represents educators’ race/ethnicity, educational attainment, title, salary, and years of working experience. STS represents secondary traumatic stress, Emo represents emotional exhaustion, Com represents compassion satisfaction, and Dis represents workplace discrimination. The term $e_{\mathrm{ij}}\;$ represents a normally distributed error term [ $e_{\mathrm{ij}}$ ~N (0, $\sigma^{2}$ )]. The terms $\mu_{0j}$ represent normally distributed variances at the center level [ $\mu_{0j}$ ~ *N* (0, $\tau_{\beta }$ )], respectively. We allowed intercepts to vary randomly at the teacher level (T = [ $\tau_{00}$ ]).

Finally, to examine the underlying reasons behind Head Start Educators’ turnover intentions, we counted the frequency of turnover intention reasons educators selected among the 13 options. Additionally, we coded participants’ answers to the “Other” option in the question and included the frequency of common answers (*n* > 2) regarding their potential turnover intention reasons.

## Results

Table 2 presents the descriptive statistics and bivariate correlations between key variables. Educators’ STS was significantly correlated with their intention to leave the profession, program, and position. Similarly, emotional exhaustion was positively significantly correlated with educators’ intention to leave the ECE profession, program, and current position. Educators’ compassion satisfaction was negatively correlated with their intentions to leave the profession, program, and position. Perceived workplace discrimination was significantly correlated with educators’ turnover intentions regarding profession, program, and position. Additionally, educators’ perceived workplace discrimination demonstrated significantly positive correlations with their STS, emotional exhaustion, and negative correlation with compassion satisfaction.

**Table 2:** Descriptive statistics and bivariate correlations.

Key variables	1	2	3	4	5	6	7
1. Intention to leave the profession	-						
2. Intention to leave the program	0.54***	-					
3. Intention to leave the position	0.30***	0.38***	-				
4. Secondary traumatic stress	0.14*	0.20***	0.14*	-			
5. Emotional exhaustion	0.23***	0.35***	0.34***	0.50***	-		
6. Compassion satisfaction	−0.35***	−0.32***	−0.19**	−0.35***	−0.37***	-	
7. Perceived workplace discrimination	0.22***	0.33***	0.16**	0.33***	0.27***	−0.35***	-
*N*	276	273	285	304	283	274	285
Mean	1.71	1.82	2.68	7.43	2.12	4.23	2.01
Standard Deviation	1.09	1.07	1.18	7.18	1.38	0.70	0.76
Range	1–5	1–5	1–4	0–36	0–5.3	1–5	1–5

*p < 0.05; **p < 0.01; ***p < 0.001.

The ICCs indicate that 0.08, 2.70, and 4.90% of the variance existed between educators within centers in educators’ intentions to leave the profession, program, and position, respectively. Table 3 shows the results from the primary effects models. First, workplace discrimination ( β = 0.12, *SE* = 0.06, *p* = 0.04) and compassion satisfaction ( β = −0.30, *SE* = 0.07, *p* < 0.00) were associated with educators’ intention to leave the ECE profession. This suggests that one standard deviation increase in compassion satisfaction is associated with a 0.30 standard deviation decrease in intention to leave the profession after controlling for other variables. In addition, emotional exhaustion ( β = 0.24, *SE* = 0.07, *p* = 0.001) and compassion satisfaction (*β* = −0.22, *SE* = 0.07, *p* = 0.002), and workplace discrimination ( β = 0.21, *SE* = 0.06, *p* = 0.001) were significantly related to educators’ intention to leave their current program. These suggest that one standard deviation increase in emotional exhaustion is associated with a 0.24 standard deviation increase in intention to leave the program, controlling for other variables. A one standard deviation increase in compassion satisfaction is associated with a 0.22 standard deviation decrease in intention of leaving the program after controlling for other variables. A one standard deviation increase in workplace discrimination is associated with a 0.21 standard deviation increase in intention to leave the program after controlling for other variables. Furthermore, emotional exhaustion ( β = 0.34, *SE* = 0.07, *p* < 0.001) was significantly associated with educators’ increased intention to leave their current position. This suggests that a one standard deviation increase in emotional exhaustion is associated with a 0.34 standard deviation increase in intention to leave the position, controlling for other variables. In comparison to the baseline (intercepts only) model, the primary models explained: (a) 2.20% of the teacher-level variance and no center-level variance in intention to leave the profession, (b) 22.701% of the teacher-level variance and 1.60% of the center-level variance in intention to leave the program, and (c) 15.85% of the teacher-level variance and 0.03% of the center-level variance in intention to leave the program^1^. Workplace discrimination did not moderate the relationships between the three key independent variables (i.e., STS, emotional exhaustion, and compassion satisfaction) and their turnover intentions.

**Table 3:** Multilevel multiple regression examining the main effects with imputed data.

	Intention to leave the profession				Intention to leave the program				Intention to leave the position			
Main effects model fixed effects	β	SE	CI	B	β	SE	CI	B	β	SE	CI	B
Secondary traumatic stress	−0.09	0.08	[−0.05, 0.07]	−0.01	−0.11	0.07	[−0.24, 0.03]	−0.02	−0.05	0.07	[−0.18, 0.08]	−0.01
Emotional exhaustion	0.11	0.07	[−0.03, 0.24]	0.08	0.24***	0.07	[0.10, 0.37]	0.18***	0.34***	0.07	[0.20, 0.47]	0.28***
Compassion satisfaction	−0.30***	0.07	[−0.43, −0.17]	−0.47***	−0.22***	0.07	[−0.35, −0.08]	−0.33**	−0.04	0.07	[−0.17, 0.10]	−0.07
Workplace discrimination	0.12*	0.06	[−0.01, 0.24]	0.17*	0.21***	0.06	[0.09, 0.34]	0.30***	0.05	0.06	[−0.07, 0.17]	0.09
Covariates												
Race/Ethnicity												
Black, non-Hispanic	−0.15	0.17	[−0.48, 0.18]	−0.19	−0.29	0.16	[−0.61, 0.03]	−0.30	0.30	0.17	[−0.03, 0.62]	0.35
Hispanic	0.04	0.18	[−0.32, 0.40]	0.10	−0.05	0.19	[−0.42, 0.33]	−0.00	0.24	0.19	[−0.13, 0.62]	0.22
Other races	−0.09	0.30	[−0.67, 0.50]	−0.21	−0.29	0.29	[−0.86, 0.28]	−0.35	0.22	0.30	[−0.38, 0.81]	0.02
Salary	0.02	0.06	[−0.10, 0.15]	0.02	0.00	0.06	[−0.12, 0.13]	0.01	−0.14	0.07	[−0.27, 0.01]	−0.06
Years of working experience	−0.06	0.06	[−0.18, 0.06]	−0.03	−0.06	0.06	[−0.05, 0.17]	0.01	−0.05	0.06	[−0.17, 0.07]	0.00
Education attainment	−0.02	0.06	[−0.13, 0.10]	−0.03	−0.01	0.06	[−0.13, 0.10]	−0.00	0.02	0.06	[−0.09, 0.13]	0.10
Title												
Assistant teacher	−0.13	0.14	[−0.40, 0.14]	−0.14	−0.09	0.14	[−0.37, 0.19]	−0.10	−0.32	0.14	[−0.60, −0.04]	−0.36
Administrative roles	0.54***	0.16	[0.22, 0.86]	0.59***	−0.01	0.16	[−0.34, 0.31]	−0.02	−0.07	0.17	[−0.39, 0.26]	0.14
Other ECE educator roles	0.36*	0.17	[0.02, 0.69]	0.39*	0.15	0.90	[−0.18, 0.49]	0.16	−0.52	0.18	[−0.87, −0.18]	−0.24
Random effects												
Center (Level 2, τ_π_)	0.00 (0.00)				0.17 (0.11)				0.01 (0.04)			
Teacher (Level 1, σ^2^)	0.96 (0.04)				0.92 (0.04)				1.08 (0.05)			
Explained variance												
Level 2	0.00%				1.60%				0.03%			
Level 1	22.20%				22.70%				15.85%			
Model fit indices												
AIC	877.1980				850.2309				941.1964			
BIC	936.6705				909.7033				1000.669			
Deviance	845.1980				818.2308				909.1964			

β = standardized coefficients; B = unstandardized coefficients; SE = standard errors, CI = 90% confidence interval; explained % is a comparison to the baseline model. *p < 0.05; **p < 0.01.; ***p < 0.001.

Table 4 presents the reasons for educators to consider turnover. Among Head Start educators who planned to leave within the next two years, a significant majority identified the desire for improved benefits and compensation as a key factor. Additionally, many educators expressed interest in exploring job opportunities outside the ECE field or transitioning to different roles within ECE. Stress related to classroom management and lack of flexibility were also common reasons for considering turnover. The pursuit of career advancement opportunities and promotions was another commonly mentioned factor. A smaller proportion of educators reported personal reasons, such as relocation or retirement, as their reasons for leaving their jobs.

**Table 4:** Reasons for educators’ turnover intentions.

Turnover intentions reasons	*N*
I want a higher paying job.	45
I want better benefits.	24
I am looking for a different job opportunity outside of early care and education.	21
There are no career advancement opportunities for me in my current program.	21
I would like to teach in a school-based program, including K-12.	16
I would like to transition to a different position in the early childhood field (e.g., home visiting, early intervention, etc.)	15
I want a job that has more flexibility (e.g., working different or fewer hours).	13
I plan to receive a promotion in my current program.	12
Retirement	9
I plan to move.	8
I would like to teach in a different early childhood program.	8
Classroom management is stressful.	7

The question was only presented to participants who indicated that they would leave their present position within their program in two years or less (*n* = 94).

## Discussion

Given the significant turnover rates among Head Start educators (Jeon and Wells, 2018; Wells, 2015) and their crucial roles in supporting children and families in need (Jeon et al., 2014), the current study examined the factors related to their turnover intentions. Our findings suggest significant associations between professional well-being and intentions to leave. Additionally, more frequent perceived workplace discrimination was found to be associated with a higher likelihood of leaving the profession or program. Overall, the findings are aligned with the Ecological Model of Holistic Early Childhood Workforce Well-being (Jeon et al., 2022), suggesting that professional well-being and positive work environment represent critical individual-level and organization-level resources that can be beneficial for educators’ commitment and job attitudes.

### Secondary traumatic stress and turnover intentions

Contrary to our hypothesis, we found no significant associations between STS and educators’ intentions to leave the profession, program, or position. Although STS is a concern among general school teachers, it remains understudied in the ECE field (Sprang et al., 2011; West et al., 2018). It is noted that Head Start educators, on average, reported relatively low levels of STS with limited variability in our study. In addition, our samples on average had over 11 years of experience in ECE, which may reflect their accumulated coping resources that buffer the direct associations between STS and job attitudes. Furthermore, it is possible explanation is that educators entering Head Start programs are often aware of the potential for exposure to STS due to the nature of working with vulnerable children. These teachers are likely motivated to care for young children who are at higher risk of experiencing traumatic experiences (Bullough et al., 2012). As they become more aware of the complexities of these children’s lives and their families’ situations, they may still feel motivated to continue their job regardless of the potential risks of experiencing STS. Because STS has been found to impact individuals’ general well-being and health (Hydon et al., 2015), despite the findings on the insignificant role of STS, future research with larger and more diverse samples of ECE professionals could further examine the relationship between STS and turnover intentions.

### Emotional exhaustion and turnover intentions

As expected, our study demonstrates a positive association between Head Start educators’ emotional exhaustion and their intentions to leave their current program and position. This suggests that educators experiencing higher levels of emotional exhaustion were more likely to consider leaving their current role and potentially transitioning to a different program. This finding aligns with existing research highlighting the prevalence of emotional exhaustion among ECE educators, often related to factors such as workload pressure, low income, and lack of resources (Al-Adwan and Al-Khayat, 2017).

However, unlike previous research suggesting a significant association between emotional exhaustion and quitting the field altogether among ECE educators in diverse types of programs (Grant et al., 2019), we did not find this association in the current study within the Head Start context. This finding also differed from non-U.S. studies. For instance, a meta-analysis study on Chinese preschool teachers found that approximately 57% of these teachers reported high levels of emotional exhaustion, which is one of the most critical factors associated with turnover intention (Xu et al., 2023). This may be related to the unique sample of Head Start educators. These educators are often driven by strong intrinsic motivations to support vulnerable children and families (Bullough et al., 2012). While emotional exhaustion may be negatively associated with their current position in the program, it may not lead them to abandon the field entirely. Instead, they might seek a change within the Head Start system, such as transitioning to a different program or altering their role within the current program, allowing them to continue working with young children in a less demanding environment.

### Compassion satisfaction and turnover intentions

Partially consistent with our hypothesis, the study demonstrated the negative association between educators’ compassion satisfaction and their intentions to leave the ECE profession and the current Head Start program within a year. This result is aligned with the prior study indicating that educators who experience lower levels of compassion satisfaction are more likely to consider quitting the education field (Christian-Brandt et al., 2020). By contrast, individuals with higher levels of compassion satisfaction are more likely to be committed to their organization (Vagharseyyedin et al., 2018), which is related to a lower likelihood of leaving their organizations (Grant et al., 2019). Interestingly, our study found that Head Start educators in our sample reported relatively high levels of compassion satisfaction, with a mean of 4.23 in the range of 1–5. This suggests strong intrinsic motivation among these educators, and those experiencing a greater sense of fulfillment from helping children in need were more likely to be committed to both their program and the broader ECE field. The findings are also aligned with evidence from studies in Finland and Ireland ECE teachers, demonstrating that compassion and compassion satisfaction are beneficial for teachers’ educational practices and serve as a protective factor for their job attitudes (O’Toole and Dobutowitsch, 2023; Vuorinen et al., 2021).

However, compassion satisfaction was not significantly associated with educators’ intentions to leave their current positions. One potential explanation is that compassion satisfaction might primarily reflect an individual’s sense of fulfillment derived from helping others within a specific profession (Stamm, 2009) or organization (Vagharseyyedin et al., 2018). It might not directly translate to satisfaction with the specific features of their current position. This finding suggests a distinction between the role of compassion satisfaction in educators’ broader professional identity and organizational commitment, versus their satisfaction with the immediate job details. Future research could examine this possibility by examining the relationship between compassion satisfaction and turnover intentions across different contexts and timeframes.

### Workplace discrimination, professional well-being, and turnover intentions

We found a significant association between perceptions of workplace discrimination and an increased likelihood of educators leaving the ECE profession and their current program. This aligns with existing research, which indicates a direct relationship between educators’ satisfaction in the workplace environment and turnover (Jeon and Wells, 2018). However, it is important to note that the assessment of workplace discrimination in this study focused on the broader workplace environment rather than on specific job positions. Thus, it is reasonable that educators perceived discrimination was predominantly associated with their intentions to leave their current program and the ECE profession rather than their particular position. The findings are supported by the Self-Determination Theory (Ryan and Deci, 2000) as discrimination may create relational and cultural demands, serving as a barrier for educators to meet basic needs for relatedness and belonging at the workplace, which may further relate to their higher turnover intention to leave the ECE field and program. In addition, this aligns with the fact that ECE educators often face similar challenges related to their professional recognition and societal respect regardless of their position (Boyd, 2013; Boyd-Swan and Herbst, 2019; Liu et al., 2025). In particular, the perception of discrimination can be prevalent in the ECE field because their work is often stereotyped as babysitting (Boyd, 2013). This systemic issue may lead to a discriminatory perception regardless of an individual’s specific role within the program. This finding suggests the importance of systematic and program-level efforts to foster an inclusive and equitable work environment, free from prejudice and discrimination, to enhance educators’ motivation to remain in their program and the profession.

However, the finding did not support our hypothesis that educators’ perceived workplace discrimination moderates the associations between the indicators of their professional well-being and their turnover intentions. One of the explanations is that compared to the specific workplace discrimination educators experienced at the workplace, professional well-being might be a more fundamental factor related to turnover intentions, as the connections suggested in existing studies (Schaack et al., 2020). Therefore, educators with high emotional exhaustion or low compassion satisfaction were likely to consider leaving regardless of the discrimination they faced. Second, the relatively low reported levels of discrimination with high variance in our sample might have limited the statistical power to detect a significant moderating effect. Third, the hypothesized moderation model may reflect a conceptual misalignment, where workplace discrimination may not necessarily weaken or strengthen the relationships between professional well-being and turnover intentions; it may only serve as an independent predictor within the Head Start context. Future research may test alternative mechanisms within larger and more diverse samples of Head Start educators to better understand the role of workplace discrimination in the associations between professional well-being and turnover intentions.

### Reasons for turnover intentions

In our exploration of the reasons behind Head Start educators’ turnover considerations, compensation and benefits were identified as the primary factors influencing their decision to leave their positions. The desire for higher pay and improved benefits was frequently chosen within Head Start educators as key motivators of turnover. This finding is consistent with prior research indicating widespread dissatisfaction among educators regarding their compensation (Bullough et al., 2012; Zhao and Jeon, 2024). Existing studies also suggest the necessity of adequate rewards for the long-term sustainability of the ECE profession (Thorpe et al., 2020).

It is well known that ECE educators receive low compensation which is often cited as a common stressor (McLean et al., 2021). However, poor salaries and compensation were not the only reasons behind turnover decisions; limited opportunities for career advancement and promotion were also identified as significant contributors to educators’ intentions to leave. This aspect has often been neglected in professional development within the ECE field (Schachter et al., 2019). Research on early childhood educators indicates that those with advanced qualifications, such as higher education degrees, tended to show a greater intention to either leave their present ECE positions or seek advancement into managerial roles (Thorpe et al., 2020). This preference is driven by the enhanced salary, autonomy, and career status that management positions offer compared to non-managerial roles (Thorpe et al., 2020). Therefore, early childhood programs should provide more advancement opportunities to help retain educators by recognizing their qualifications and offering them a path for professional growth.

Furthermore, we found that the challenges associated with classroom management and inflexible working schedules were also the reasons influencing educators’ decisions to potentially quit their roles. Head Start educators face more challenges and want to receive additional resources to manage children’s challenging behaviors (Zhao and Jeon, 2024). Previous research indicates that some Head Start educators reported that they do not even have enough bathroom breaks and have a hard time balancing their lives and work due to the inflexible working schedule (Wang et al., n.d.). The focus of the improvement of the ECE workforce quality has predominantly been on enhancing professionalism via training and accountability measures. However, this has not been adequately matched with social recognition and support for educators (Schachter et al., 2019).

### Limitations

While this study provides insights into Head Start educators’ well-being and turnover intentions, there are several limitations within this study. First, we used cross-sectional data and correlational modeling, which limited our ability to establish causality and/or directionality for the relationships among key variables. It is noted that all significant relationships discovered in this study might be reciprocal or bidirectional. It is possible that the observed associations may exist in the reverse direction from what we hypothesized. For example, it could be that educators with a higher intention to leave the profession perceive less compassion satisfaction in their work. This research, therefore, lays the foundation for future investigations to examine the directional nature of these relationships.

Second, we collected teachers’ self-reported data to gather information on teachers’ perceptions of their well-being and workplace environment. This may be subject to both social desirability bias and construct validity. While this approach offers insight into the educators’ perspectives, which is crucial for understanding their attitudes and behavior (Cohen et al., 2016), future research could incorporate additional data types. For example, these could include observational data on workplace climate and administrative and longitudinal data on actual turnover behavior, which can ensure the predictive power of the study and provide a more comprehensive understanding beneficial for ECE policymakers and administrators. Future research can also examine contextual variables, such as the urbanicity of the program, level of funding for programs, and ethnicities in leadership positions, that potentially impact educators’ work experiences and attitudes. Third, we used single items for the measures of turnover intentions in order to reduce cognitive burden and response fatigue for educators. While research shows that single-item measures can also be valid and reliable when measuring concrete and unambiguous constructs (Nagy, 2002; Siebens et al., 2015), it still limits our ability to triangulate individuals’ intentions through multiple items. We recommend that future research also explore multi-item scales to capture each type of turnover intention within the ECE workforce population.

Moreover, the generalizability of the findings of this study is limited. Our focus was specifically on educators within center-based Head Start programs, aiming to contribute to the development of the Head Start workforce. However, these findings may not fully represent the experiences of ECE educators in other contexts, such as private or family childcare settings. Future research should consider examining the associations between professional well-being and turnover intentions across a broader range of ECE environments to enhance the applicability and impact of the findings in the field.

### Implications

Despite the limitations, the study provides implications for researchers, policymakers, and practitioners in the ECE field. First, while there is existing research on the well-being of ECE educators, such as stress and depressive symptoms, and their associations with turnover intentions (e.g., Bryant et al., 2023; Grant et al., 2019), our study extended the literature by examining the relationships between STS, emotional exhaustion, compassion satisfaction, workplace discrimination, and various types of turnover intentions among Head Start educators. These factors (e.g., STS and compassion satisfaction), although widely investigated in other settings (e.g., the healthcare field) have received less attention in the context of Head Start programs. Due to the distinct work environment of Head Start educators who are working with children with a higher risk of experiencing traumatic events (Rankin, 2021), their compassion satisfaction can become a protective factor and motivate them to provide more support for children and families in most need (Fleckman et al., 2022). Our findings suggest the importance of these factors in the ECE field, especially in Head Start settings. Future studies can expand upon this by investigating these factors in larger and more representative samples to better understand their impact on actual turnover behaviors.

Second, the study provides actionable insights for Head Start administrators. It suggests that enhancing educators’ compassion satisfaction may be key to reducing turnover intentions at both the program level and across the profession. Although research on compassion satisfaction among early childhood teachers is limited, insights can be gleaned from studies in the healthcare sector. Strategies like reflective supervision and robust managerial support have been shown to increase compassion satisfaction in healthcare professionals (Hunsaker et al., 2015). In addition, when healthcare workers feel more equipped to deal with the challenges at work, they are more likely to have higher levels of compassion satisfaction (Al-Otaibi and Kerari, 2025). Similarly, providing Head Start educators with adequate support and guidance in managing challenges such as children’s difficult behaviors, classroom management, and emergency situations could be potentially related to their increased compassion satisfaction. Furthermore, Morera et al. (2025) suggest that more organizational support and meaningful recognition of efforts are crucial for increasing compassion satisfaction. This is particularly crucial in the ECE, where educators often experience low societal recognition evidenced by low salaries and a lack of social recognition, for their work (Sparks, 2019). Therefore, systematic and organizational changes are needed regarding increasing compensation and demonstrating societal values for Head Start educators’ work in order to increase their compassion satisfaction and, ultimately, reduce their intent to leave.

Third, our study suggests the importance of reducing emotional exhaustion among Head Start educators to sustain their engagement in their roles. For example, mindfulness-based interventions have been found to be effective in reducing overall stress and emotional exhaustion in ECE teachers, particularly for those working with children experiencing trauma and adversity (Jennings, 2015; Taylor et al., 2021). Thus, Head Start programs may consider incorporating mindfulness training in their professional development and routines (e.g., breathing activities). Emotional exhaustion is often related to overwhelming workloads and is frequently viewed as an occupational hazard in the teaching profession (Al-Adwan and Al-Khayat, 2017). Encouragingly, research indicates that increased social support, especially supportive colleague relationships, is associated with reduced emotional exhaustion levels in Head Start educators (Song et al., 2020). Furthermore, reducing educator emotional exhaustion also involves empowerment and support from leadership. Literature in emotional literacy suggests that improving educators’ emotional competence may be beneficial for their improved compassion and reduced emotional exhaustion, especially for educators who work with students from marginalized backgrounds (Calandri et al., 2025). These can include strategies such as providing more autonomy, offering well-being resources focusing on emotional competence, delivering professional development in inclusive and trauma-informed pedagogy, and enhancing teachers’ involvement in decision-making processes (Calandri et al., 2025; Sandilos et al., 2024). These measures not only address immediate stressors but also contribute to a more supportive and fulfilling work environment, which can benefit both educators and the children in their classroom.

Fourth, this study demonstrates the associations between workplace discrimination and turnover intentions of Head Start educators. Particularly for those working with children and families from disadvantaged backgrounds, perceptions of prejudice and discrimination within their work environment can have direct consequences on the engagement and development of the children they teach (Butler-Barnes et al., 2022). It is critical for policymakers and program leaders to identify and address the root causes of workplace discrimination at both systematic and institutional levels. Systematically, ensuring equitable compensation for Head Start educators in line with their counterparts in other ECE settings is a crucial step (McLean et al., 2021). In addition, a critical review and revision of hiring practices are necessary to promote equity and reduce discrimination, including measures to increase staff diversity and achieve racial/ethnic congruence between teachers and students (Dhanani et al., 2018). At the program level, the recruitment of experts and leaders committed to equity and diversity is essential for establishing a long-term strategy to minimize discrimination within these programs (Joseph, 2021).

Fifth, in terms of the specific reasons behind educators’ turnover, our study demonstrated that inadequate salary and benefits are the primary factors driving Head Start educators to consider leaving their jobs, aligning with findings from previous research in the field of ECE (Schaack et al., 2022). However, these are not the only concerns. There was also a clear need for more comprehensive support and training in career advancement, including opportunities for promotion. Current professional development programs tend to focus on enhancing teaching skills for children’s learning and development, rather than addressing the career aspirations of the educators themselves (Schachter et al., 2019). Therefore, it is crucial for Head Start program leaders to provide resources and support for educators’ career development, actively involving them in decision-making processes, and preparing them for potential leadership roles (Kirby et al., 2021). Additionally, stress related to classroom management and a desire for different roles within the educational sector were also reasons for turnover intentions. Some educators expressed interest in transitioning to roles like home visiting in early childhood programs or teaching in K-12 settings. These may be due to the challenges faced by Head Start educators, including the need for additional support and resources to manage children’s challenging behaviors (Kwon et al., 2022b; Zhao and Jeon, 2024). To reduce actual turnover, it is critical for policymakers and Head Start administrators to understand these underlying reasons and provide additional resources that address these stressors and challenges, thereby retaining high-quality educators in these crucial roles.

## Conclusion

Head Start workforce is the foundation of high-quality education for young children who experience poverty and more adversities than their peers (Jeon and Wells, 2018). Therefore, it is important to support the Head Start workforce and improve their professional attitudes in order to help children in need. The current study suggests that supporting these educators through fostering compassion satisfaction, reducing emotional exhaustion, and cultivating an environment that prioritizes equity can contribute to retaining a high-quality Head Start workforce. In addition, addressing financial burdens and providing career advancement opportunities are essential to address the ongoing teacher shortage crisis and ensure Head Start program sustainability.

## Data Availability

The datasets presented in this article are not readily available because due to the inclusion of sensitive private mental health-related information about participants, the data is not publicly available at this moment. Requests to access the datasets should be directed to XZ, kvs4ft@virginia.edu.

## Footnotes

^1^In addition to our primary analyses, we conducted a sensitivity analysis to assess the impact of including a dummy variable for the grantee in each model. However, including the grantee variable had no significant effect on the relationships observed among the variables. Given these findings and the principle of parsimony, we excluded the grantee variable from the final models.
